# A Simplified Tea Ceremony Experience for Palliative Care Staff: A Feasibility and Acceptability Study

**DOI:** 10.7759/cureus.100865

**Published:** 2026-01-05

**Authors:** Shuku Nishikori, Ai Taruoka, Aki Kajita, Susumu Joyama, Eriko Sugano, Yuko Kamiya, Kazuyuki Niki, Makie Kohno, Yoshinobu Matsuda, Ryouhei Ishii

**Affiliations:** 1 Department of Rehabilitation, Ashiya Municipal Hospital, Ashiya, JPN; 2 Department of Palliative Medicine, Ashiya Municipal Hospital, Ashiya, JPN; 3 Center for Advanced Education and Research in Pharmaceutical Sciences, Graduate School of Pharmaceutical Sciences, The University of Osaka, Suita, JPN; 4 Psychiatry, The University of Osaka, Suita, JPN

**Keywords:** anxiety levels, japanese powder tea, mindfulness, nurses' occupational stress, occupational therapy, supportive and palliative care

## Abstract

Background: Palliative care staff experience high occupational stress, yet brief, culturally informed interventions remain underexplored. The Japanese tea ceremony integrates ritual, mindfulness elements, and sensory engagement, but its feasibility and acceptability in healthcare settings are unknown. The aim of this study was to assess the feasibility and acceptability of implementing a simplified tea ceremony intervention for palliative care staff, and to explore participants' subjective experiences to inform future controlled research.

Methods: Ten palliative care staff members from a single institution participated in a 20-minute simplified tea ceremony. Feasibility was assessed through recruitment and completion rates. Acceptability was evaluated through qualitative feedback, while changes in psychological state were descriptively assessed using the State-Trait Anxiety Inventory (STAI) administered pre- and post-intervention. Descriptive statistics characterized pre-post changes in self-reported anxiety; no inferential statistics were used, as this study was not designed to test efficacy hypotheses.

Results: Recruitment and retention were 100%, indicating feasibility. Mean state anxiety scores decreased from 51.5 (SD=7.0) to 44.2 (SD=6.9), while trait anxiety decreased from 46.8 (SD=6.7) to 43.7 (SD=5.9). Qualitative feedback revealed three themes: immersion in ritual (8/10 participants), sensory calmness (9/10 participants), and positive distraction (10/10 participants). Two participants noted discomfort with formality.

Conclusion: A simplified tea ceremony is feasible and acceptable for palliative care staff. Participants reported positive subjective experiences and temporary reductions in self-reported anxiety. However, this study provides no evidence regarding the tea ceremony's specific therapeutic efficacy, as the design cannot distinguish its effects from rest, attention, or expectancy. Randomized controlled trials comparing the tea ceremony to active and passive control conditions are needed.

## Introduction

Healthcare professionals working in palliative care settings face chronic occupational stressors, including repeated exposure to patient suffering, death, moral distress, and emotional labor. These stressors contribute to burnout, compassion fatigue, and reduced well-being, with documented prevalence rates of 30%-60% in this population. Effective, accessible stress-reduction interventions are urgently needed. Mindfulness-based interventions (MBIs), such as Mindfulness-Based Stress Reduction (MBSR), have demonstrated efficacy in reducing stress and anxiety among healthcare workers in multiple systematic reviews and meta-analyses. Brief mindfulness interventions, typically under 30 minutes, have also shown promise, though often with small to moderate effect sizes (d=0.2-0.5) [1-6].

A critical gap exists in understanding whether the combined multi-modal effects of the tea ceremony exceed those from rest or other brief interventions. The Japanese tea ceremony (chanoyu) is a formalized ritual practice combining prescribed movements, aesthetic contemplation, social interaction, and consumption of matcha (powdered green tea). Unlike secular mindfulness training, the tea ceremony is embedded in cultural tradition and combines multiple potentially therapeutic components. Psychological research suggests that ritualized behaviors can reduce anxiety through an increased sense of control, predictability, and meaning-making, though evidence is mixed regarding whether formality enhances or impedes relaxation. The ceremony also requires present-moment focus on sensory details, sharing features with mindfulness meditation but differing in its external, social, and cultural framing. Matcha itself contains L-theanine, an amino acid that modulates GABA (gamma-aminobutyric acid), dopamine, and serotonin neurotransmission. However, clinical trials showing modest anxiolytic effects have used L-theanine doses of 200-400mg, which is substantially higher than the 20-40mg found in a standard bowl of matcha, making direct biochemical effects uncertain. The ceremony also involves shared ritual and gentle interaction, which may provide social support benefits, though no studies have isolated this component [7-10].

Shimizu et al. (2016) reported that a tea ceremony program improved subjective mood and reduced pulse rate in a psychiatric daycare setting. However, their uncontrolled study could not distinguish specific effects of the tea ceremony from time, rest, or social interaction [11]. No studies have examined tea ceremony interventions in palliative care staff or compared them to alternative brief interventions. Given the absence of foundational research on tea-ceremony-based approaches for palliative care staff, this pilot study aimed to (1) assess the feasibility of recruiting palliative care staff and delivering a 20-minute simplified tea ceremony and (2) assess acceptability through participant feedback and pre-post descriptive measures to inform future controlled trials. This study is explicitly not designed to test the efficacy of the tea ceremony but to determine if a future randomized controlled trial is justified.

## Materials and methods

This single-arm feasibility and acceptability study with pre-post measurement used an observational and descriptive design; no causal inferences were intended. Participants were recruited from Ashiya Municipal Hospital, a secondary emergency hospital in Hyogo, Japan. This site was selected based on the researchers' affiliation and accessibility for this pilot feasibility study. Inclusion criteria were as follows: (1) current employment as medical staff in the palliative care unit, and (2) willingness to participate in the tea ceremony. Exclusion criteria were as follows: (1) allergy to matcha (green tea) or its ingredients, and (2) physical inability to sit in a Japanese-style room for 20 minutes. Ten staff members from the palliative care unit at the Ashiya Municipal Hospital volunteered after responding to recruitment materials. The first author (S.N.) provided a short verbal explanation of the study using a simple flyer during a regular staff meeting and invited interested staff to contact the research team directly. All participants were directly involved in palliative care and gave written informed consent. The sample included four occupational therapists, two nurses, one clinical psychologist, one pharmacist, and two physicians (six women and four men; mean age, 36.2 years, SD=8.4). The sample size was not determined by statistical power analysis, as this was a pilot feasibility study. Instead, the sample size was determined based on the availability of eligible staff members in the palliative care unit during the study period. This small sample size is insufficient for hypothesis testing, and any statistical estimates are intended for descriptive purposes only. The study was conducted between April 2025 and October 2025.

Participants attended a single 20-minute simplified tea ceremony in a quiet Japanese-style room (tatami room) located within the hospital. To minimize confounding olfactory factors, no incense or artificial room perfumes were used; the aroma was limited to the natural scent of the matcha tea. The session was guided by the first author (S.N.), who has 10 years of experience practicing the Japanese tea ceremony. The traditional ceremony was simplified by removing strict etiquette requirements for guests (e.g., specific bowing angles, and rotation of the bowl) and using a table-and-chair style (ryurei) for participants unable to sit in the seiza position, while retaining the core sequence of purifying utensils and whisking tea to maintain ritual integrity. Participants were invited to focus on specific sensory experiences, such as the visual appearance of the tea utensils and the green color of the matcha, the sound of the bamboo whisk in the bowl, the aroma rising from the tea, the taste of the matcha and the accompanying sweet, and the warmth and texture of the tea bowl in their hands, without any performance pressure. The session involved a demonstration of matcha preparation, self-preparation of matcha with a traditional sweet, and silent consumption, with the facilitator occasionally directing attention to sensory details.

Feasibility was measured by recruitment and completion rates. For acceptability, quantitative data were collected using the Japanese version of the State-Trait Anxiety Inventory (STAI), which differentiates state anxiety from trait anxiety on a scale of 20-80. The STAI was administered immediately before the tea ceremony, after participants had been seated in the Japanese-style room, and again immediately after the 20-minute session, in the same room, before they returned to clinical duties; thus, the interval between pre- and post-measurements was approximately 20-25 minutes. The Japanese version of the STAI has demonstrated good psychometric properties, including high internal consistency (Cronbach’s alpha values typically exceeding 0.85 for both state and trait scales) and acceptable construct validity in clinical and non-clinical samples, supporting its use for assessing anxiety in Japanese populations [12].

The purpose of this pre-post measurement was not to test effectiveness, but to (1) assess the acceptability of the measurement procedure itself and (2) generate preliminary descriptive data to inform the sample size calculation for a future definitive randomized controlled trial. Qualitative data were collected via three open-ended questions. Participants provided their responses in writing (paper-and-pencil method) immediately following the intervention. These handwritten responses were transcribed verbatim into an electronic format by the research team prior to analysis [2]. These questions were developed specifically for this feasibility study to explore participants’ subjective impressions and were not part of a standardized or formally validated psychometric instrument. They were used as exploratory prompts rather than as a quantitative outcome measure.

Feasibility metrics were calculated as proportions. Pre- and post-intervention STAI scores were summarized using means and standard deviations, with the mean change and its 95% confidence interval calculated descriptively. No inferential statistics (t-tests, p-values) were used, as the study was not powered for hypothesis testing and could not support causal claims. Qualitative responses were coded thematically by two independent coders.

Specifically, the coding was conducted by S.N. and R.I., both with prior training and experience in qualitative thematic analysis and familiarity with working in palliative care settings. R.I. was not involved in supervising the participating staff and did not participate in the tea ceremony sessions. Eligibility criteria for serving as coders included the following: (1) a professional background in rehabilitation/occupational therapy, (2) prior experience in qualitative data analysis, and (3) familiarity with the clinical context of palliative care.

## Results

Recruitment was high, with 10 of 12 interested staff members (83%) enrolling; all enrolled participants (100%) completed the intervention, and no adverse events were reported. On average, participants reported lower state anxiety after the tea ceremony, with a mean decrease of 7.3 points. The 95% confidence interval for this change ranged from a reduction of 11.5 to 3.1 points, illustrating the imprecision of the estimate in this small sample. Trait anxiety also decreased by 3.1 points on average, though the confidence interval included zero, indicating substantial uncertainty (Table 1). These descriptive changes could reflect the tea ceremony's effects, but equally could be due to rest, expectancy, or other non-specific factors.

**Table 1:** Self-Reported Anxiety Scores Before and After Tea Ceremony Experience These are descriptive statistics only and do not support causal inferences.

Measure	Pre-Intervention Mean (SD)	Post-Intervention Mean (SD)	Mean Change (95% CI)
State anxiety	51.5 (7.0)	44.2 (6.9)	-7.3 (-11.5, -3.1)
Trait anxiety	46.8 (6.7)	43.7 (5.9)	-3.1 (-6.8, 0.6)

Qualitative analysis revealed three major positive themes. "Immersion in ritual" was reported by eight participants, who described a sense of absorption that helped them disengage from work-related thoughts. "Sensory calmness" was noted by nine participants, who found the focus on aroma, taste, and visual aesthetics to be relaxing. "Positive distraction" was mentioned by all 10 participants, who characterized the experience as a refreshing break from clinical routines. A minority theme, "discomfort with formality," was reported by two participants who felt mild anxiety about performing the ritual correctly. A total of 9 of the 10 participants expressed interest in participating in a similar activity in the future.

## Discussion

This feasibility and acceptability study demonstrates that a simplified tea ceremony intervention can be feasibly implemented in a palliative care setting, with high recruitment, perfect retention, and largely positive participant feedback. Descriptively, participants reported temporary decreases in self-reported anxiety, and qualitative data suggest the experience was perceived as calming and beneficial.

However, as intended by the study design, this study provides no evidence regarding the tea ceremony's therapeutic efficacy or specific effects. The observed descriptive changes in anxiety scores are uninterpretable from a causal perspective and could be entirely explained by rest and time away from clinical duties, expectancy and placebo effects, Hawthorne effects of being observed, demand characteristics, the novelty of the activity, or regression to the mean. Importantly, because this was a single-arm feasibility study without any control condition or blinding, these potential confounding factors were not controlled, and the pre-post STAI changes should be interpreted only as descriptive, not as evidence of a specific treatment effect. Furthermore, the use of the Trait Anxiety scale in a pre-post design over a 20-minute interval is conceptually debated, as trait anxiety is theoretically a stable personality characteristic. The observed changes likely reflect a temporary "state" fluctuation or measurement error rather than a genuine modification of the participants' trait anxiety. These preliminary data are presented solely to demonstrate the feasibility of collecting such outcomes and to provide an initial, albeit imprecise, estimate for planning a future, larger scale study. In addition, the extremely small sample size means that any effect size estimates would be severely inflated and unreliable, a consistent finding in research on small-sample studies [13-16]. Furthermore, we did not collect or analyze potentially relevant demographic variables such as years of clinical experience or socioeconomic status, which may influence baseline anxiety levels and relaxation responses; future adequately powered trials should include these factors and examine their impact using appropriate statistical models. Additionally, the qualitative data were limited to brief written responses, which may lack the depth and nuance of data collected through semi-structured interviews, potentially oversimplifying the participants' subjective experiences.

Our qualitative findings align with the observations of Shimizu et al. (2016), but both studies share the same fatal methodological flaw of lacking a control group [11]. While the literature suggests brief mindfulness interventions can have modest effects on state anxiety, these effects are often smaller when compared to active controls. The question of whether the multi-component tea ceremony offers benefits beyond a simple structured break remains unanswered. To address this, future research should employ a randomized controlled trial design with at least three arms: a tea ceremony group, an active control group (e.g., social conversation over herbal tea), and a passive control group (e.g., quiet rest). Such a study would require an adequate sample size (e.g., at least 64 participants per arm for 80% power to detect a small-to-moderate effect), objective physiological measures like heart rate variability, and longer term follow-up to assess the durability of any effects. Component analysis studies could also help isolate the mechanisms of ritual, sensory focus, and biochemistry [3]. Although this study does not provide efficacy evidence, it offers the first empirical evidence that a traditional Japanese cultural ritual is acceptable and feasible for hospital staff, thereby validating a novel, culturally grounded approach to occupational therapy interventions that warrants rigorous testing in future trials.

## Conclusions

A simplified tea ceremony intervention is feasible to implement and acceptable to palliative care staff. While participants reported positive subjective experiences, this study provides no evidence that the tea ceremony has specific therapeutic effects. The observed descriptive changes in anxiety could be attributable to numerous non-specific factors. These findings justify proceeding to a properly controlled randomized trial to determine if the tea ceremony offers unique stress-reduction benefits beyond those of a simple break from work. Clinicians and researchers should not interpret these preliminary findings as evidence of efficacy.
